# Research Progress on Chemiresistive Carbon Monoxide Sensors

**DOI:** 10.3390/nano15040303

**Published:** 2025-02-16

**Authors:** Minghui Wei, Xuerong Shi, Min Zhu, Shengming Zhang, Heng Zhang, Haiyu Yao, Shusheng Xu

**Affiliations:** School of Material Science and Engineering, Shanghai University of Engineering Science, Shanghai 201620, China; m350122104@sues.edu.cn (M.W.); m350123105@sues.edu.cn (M.Z.); m355123203@sues.edu.cn (S.Z.); m355124217@sues.edu.cn (H.Z.); m350122116@sues.edu.cn (H.Y.)

**Keywords:** chemiresistive, sensor, CO, detection

## Abstract

The development of high-performance carbon monoxide (CO) sensors is essential for protecting human health, ensuring industrial safety, and maintaining environmental well-being. Among various types of sensors, chemiresistive sensors exhibit considerable promise for real-time applications due to their operational capabilities. To achieve high performances of chemiresistive sensors, this review emphasizes various enhancement strategies, encompassing the refinement of sensing materials, the augmentation of sensor structures, and the optimization of gas recognition algorithms. Specifically, the modification techniques of sensing materials, which include the construction of heterostructures, the decoration with noble metals, surface functionalization, hetero-element-doping, and morphology engineering, are delved into comprehensively. This review provides insights into the rational design of cost-effective CO sensors.

## 1. Introduction

Carbon monoxide (CO) is a toxic gas characterized by its colorlessness, odorlessness, and non-irritating nature, typically resulting from incomplete combustion processes. High concentrations of CO can readily lead to poisoning incidents. Consequently, the development of an efficient and precise CO detection technology is paramount for safeguarding human lives [1,2,3,4,5].

**Figure 1:**
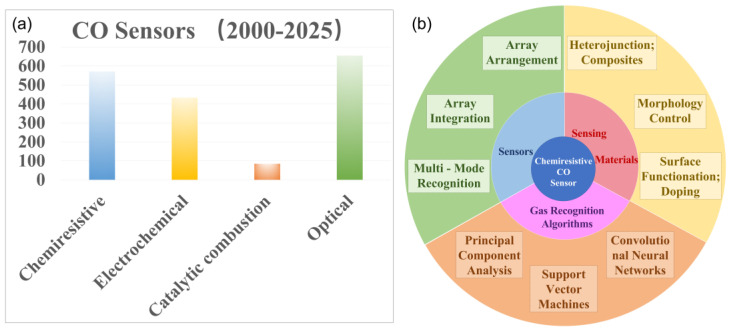
(**a**) Number of published papers and patents for various types of CO sensors from 2000 to 2025. Data from Web of Science. (**b**) Strategies employed to improve the performances of chemiresistive CO sensors.

Based on their operational mechanisms, gas sensors can be categorized into several types: (i) Chemiresistive sensors [6,7,8], which function by detecting gases through changes in the resistance of semiconductor materials. When gas molecules interact with the semiconductor surface, an oxidation-reduction reaction occurs, leading to changes in the resistance value; (ii) Electrochemical gas sensors [9,10,11], which measure gas concentrations by assessing the current produced by the oxidation or reduction in gases at an electrode [12]; (iii) Catalytic combustion gas sensors [13,14,15], which detect gas concentrations by harnessing the heat or resistance changes induced by the combustion of combustible gases on a catalyst surface; (iv) Optical gas sensors [16,17,18], which utilize optical principles for the detection and measurement of specific gas concentrations [19,20,21]. Over the past years, all of them have undergone significant developments (Figure 1a), leading to notable achievements.

Among the various gas detection technologies, chemiresistive sensors represent a prevalent methodology for detecting gases. These sensors exhibit numerous advantages, including high sensitivity, rapid response times, and cost-effectiveness, making them extensively utilized in the monitoring of diverse hazardous gases. However, they also possess certain limitations, such as insufficient selectivity, a considerable environmental impact, and elevated power consumption. For instance, when detecting gases with comparable chemical properties, such as hydrogen (H_2_) and CO, metal oxide semiconductor gas sensors exhibit similar response signals, thereby compromising the accurate identification and quantification of the target gas. Additionally, humidity also poses as a common interference. The high operating temperatures needed for optimal performance (usually 200–350°) result in high power consumption, shortening battery life in battery-powered devices, and raising costs. In contrast, reducing the power often comes at the expense of sensitivity and response speed, further limiting their use in portable and low-power applications.

To enhance the performances of chemiresistive CO sensors, various strategies have been employed, such as the optimization of sensor materials, sensor structures, signal processing methodologies (Figure 1b), etc. [22,23,24]. This review focuses on recent advancements in improving CO detection ability through the optimization of sensor materials, the enhancement measures grounded in sensor structural design, and the improvement of gas recognition algorithms. Drawing upon these discussions, the review concludes by presenting key findings and outlining potential avenues for future research and development.

## 2. Working Principle of Chemiresistive CO Sensors

Chemiresistive sensors function to detect gas concentrations by monitoring resistance variations that result from chemical reactions or physical adsorption interactions between sensitive materials and the target gas. Notably, this resistance change bears a direct proportionality to the concentration of the gas being measured. Consequently, one can accurately ascertain the gas concentration. Typically, these sensors comprise sensitive components, such as metal oxide semiconductors or conductive polymers, along with measurement circuits [25].

**Figure 2:**
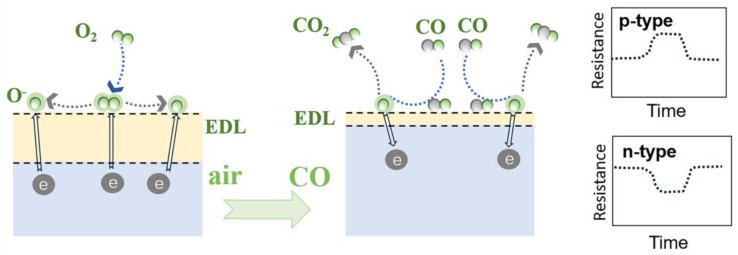
Illustration of reaction mechanism and corresponding resistance change in n-type and p-type sensors when exposed to CO.

In the presence of air, oxygen molecules first absorb on the surface of sensing materials (Equation (1)) and then draw electrons from the materials (Equation (2)), which induces an electron depletion layer (EDL) at the surface of the sensing materials, to become $O_{2}^{-}\left(\mathrm{ads}\right)$. Following this, $O_{2}^{-}\left(\mathrm{ads}\right)\;$ dissociates to generate oxygen ions $O^{-}\left(\mathrm{ads}\right)$ (Equation (3)) and gains more electrons from the sensing materials to broaden the EDL thickness (Figure 2) [26]. This electron trapping mechanism results in an augmentation of the hole concentration, thereby modulating the resistance of either n-type or p-type semiconductors (*R*_a_), with an increase or decrease, respectively. Notably, the majority of sensors discussed in this review exhibit an optimal performance at temperatures approximately 200 °C and above, where the predominantly adsorbed oxygen species are O^−^ and O^2−^ [27].

Upon exposure of the gas sensor to CO, its surface undergoes chemical adsorption of CO molecules (Equation (4)). In the presence of chemically adsorbed oxygen anions, the adsorbed CO reacts with the oxygen anions on the sensing material’s surface to form CO_2_ and release free electrons back to the materials. Consequently, the EDL thickness decreases, and it leads to an increase or decrease in the resistance of p-type or n-type sensors, respectively (Figure 2).

(1) $$O_{2}\left(\mathrm{gas}\right)\;\to {\;O}_{2}\left(\mathrm{ads}\right),$$

(2) $$O_{2}\left(\mathrm{ads}\right)+e^{-}\;\to {\;O}_{2}^{-}\left(\mathrm{ads}\right)\;,$$

(3) $$O_{2}^{-}\left(\mathrm{ads}\right)+e^{-}\;\to {\;2O}^{-}\left(\mathrm{ads}\right)\;,$$

(4) $$\mathrm{CO}\left(\mathrm{gas}\right)\;\to \;\mathrm{CO}\left(\mathrm{ads}\right)\;,$$

(5) $${\mathrm{CO}(\mathrm{ads})+O}^{-}\left(\mathrm{ads}\right)\;\to {\;\mathrm{CO}}_{2}\left(\mathrm{gas}\right)+e^{-},$$

According to the aforementioned gas-sensing mechanism, the sensor response is substantially influenced by two factors: the number of electrons captured by the adsorbed oxygen molecules within the conduction band and the number of electrons released by the target gas molecules during their reaction with the adsorbed oxygen [27,28,29,30].

**Figure 3:**
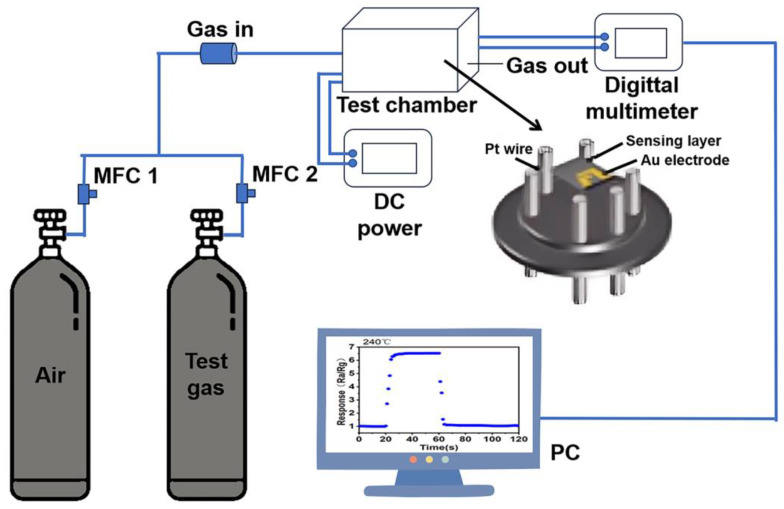
Typical CO properties testing progress [31].

A typical test process of CO sensing is illustrated in Figure 3 [31]. Alternatively, the sensing material was blended with alcohol to attain a slush-like state and then dropped onto the ceramic substrate. Subsequently, fork-finger electrodes were soldered onto the sensor’s base. Prior to conducting gas-sensitivity performance testing, the sensors underwent an aging process in air, specifically at 200 °C for two days. An electrometer was utilized to assess the sensor’s performance under both air and target gas atmospheres.

Apparently, the type and properties of sensitive materials, the concentration of CO, as well as environmental factors such as temperature and humidity collectively influence the performance of chemiresistive CO sensors. The materials employed for the sensing electrode are crucial in gas adsorption, selective catalytic reactions, and electrochemical reactions, offering sufficient sensitivity and selective responsiveness towards target gases. Common sensitive materials encompass metal oxide semiconductors (e.g., SnO_2_, ZnO, In_2_O_3_, WO_3_, CuO, Co_3_O_4_, and NiO) [32,33], conductive polymers (e.g., polyaniline, polypyrrole) [34,35], and composite materials (e.g., graphene composites) [36,37]. For example, a commercial MQ-7 CO sensor employed SnO_2_ as the sensing material. Despite significant investigations in chemiresistive CO sensors over the past few decades, there remains ample scope for enhancing sensing performances.

## 3. Improving Sensing Materials

As evident from the aforementioned discussion, the features of sensing materials, including the components, surface morphology, gas adsorption, and catalytic abilities, play a determinant role in dictating the performances of gas sensors [38,39,40,41]. Enhancing the properties of sensitive materials to augment the detection capabilities of chemiresistive CO sensors represents an efficacious strategy.

The subsequent paragraphs provide a detailed elucidation of these strategies.

### 3.1. Development of New Composite Materials

The construction of p-n heterojunctions has emerged as an efficacious approach for achieving the selective detection of reducing gases, such as H_2_ and CO [42]. Various methodologies have been proposed for the preparation of heterojunctions, encompassing the sol-gel method [43,44], hydrothermal synthesis [45,46], and chemical vapor deposition (CVD) [47,48], among others [49,50,51,52,53]. The development of heterostructures based on metal-organic framework (MOF)-derived oxides has garnered significant attention as a promising strategy to enhance sensor performances [54,55,56]. MOFs have been extensively employed as precursors or templates for the synthesis of metal oxide compounds. These frameworks consist of metal ions and organic ligands, interconnected through self-assembly to form diverse network topologies with porous structures [57,58,59]. This unique architecture imparts high porosity, a large specific surface area, and high crystallinity to MOF-derived metal oxides, which are conducive to improving gas adsorption and diffusion capabilities [60]. Consequently, MOFs and their derivatives have been broadly adopted as sensitive materials to augment the performance of gas sensors, capitalizing on their aforementioned advantages. The composition and structure of the materials can be precisely manipulated by strategically selecting appropriate metal ions and adjusting the concentrations of the precursor solutions.

**Figure 4:**
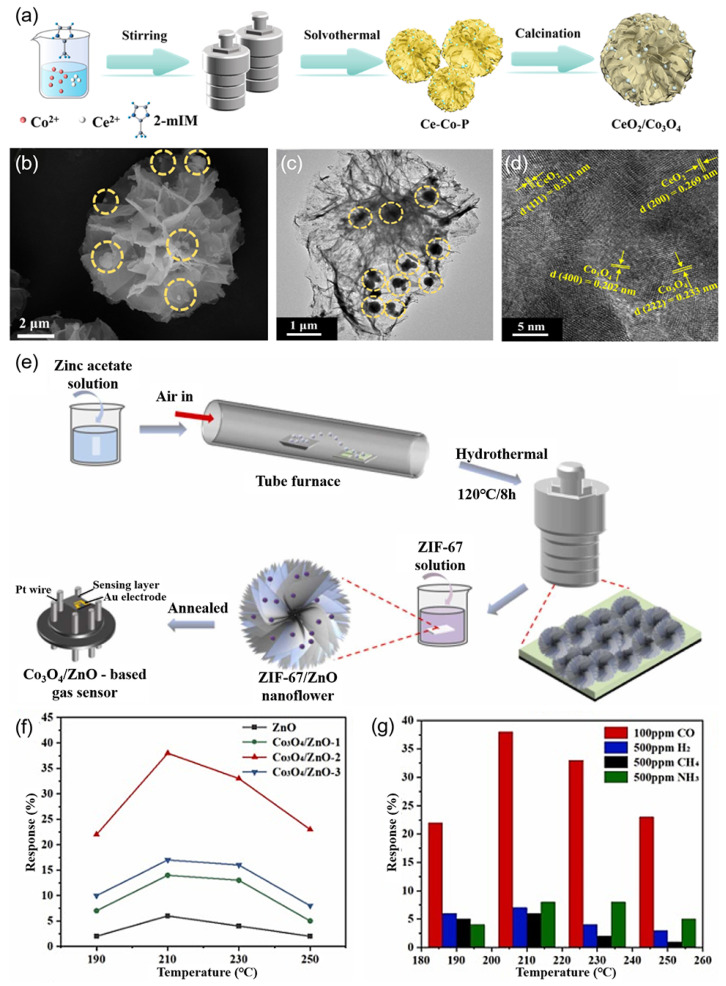
(**a**) Schematic illustration of the hierarchical CeO_2_/Co_3_O_4_ heterostructures synthesis strategy [64]. (**b**) FESEM image of 5CeO_2_/Co_3_O_4_ [64]. (**c**,**d**) TEM images of 5CeO_2_/Co_3_O_4_ [64]. (**e**) Synthesis process of Co_3_O_4_/ZnO heterojunction [31]. (**f**) Response values to 100 ppm CO vs. operating temperature and (**g**) selective response of Co_3_O_4_/ZnO-2–100 ppm CO at 210 °C [31].

Illustratively, NiO-CuO composites derived from Ni/Cu MOFs [61], NiO/FeO_x_ composites derived from Ni/Fe MOFs [62], and Co_3_O_4_/In_2_O_3_ heterostructures derived from bimetallic MOFs [63] all demonstrated significantly enhanced sensing responses towards CO. Qin et al. [64] successfully synthesized hierarchical cerium dioxide (CeO_2_)/Co_3_O_4_ heterostructures, assembled from ultrathin nanosheets (NSs), utilizing bimetallic MOFs of the ZIF-series as templates through a solvothermal-calcination procedure (Figure 4a). The CeO_2_ nanoparticles (the part circled by the yellow dotted line in Figure 4b,c) were uniformly dispersed and anchored onto the Co_3_O_4_ NSs (high-resolution TEM image of Figure 4d with distinct lattice fringes of CeO_2_ and Co_3_O_4_), with the optimal 5 mol% CeO_2_/Co_3_O_4_ (designated as 5CeO_2_/Co_3_O_4_, a remarkable specific surface area of 72.5 m^2^ g^−1^). Furthermore, the resultant 5CeO_2_/Co_3_O_4_ sensor demonstrated an exceptional response of 184% (Table 1) ((*R*_g_ − *R*_a_)/*R*_a_) to 50 parts per million (ppm) of CO at 200 °C, which represents an enhancement of approximately 4.4 times compared to the bare Co_3_O_4_-based sensor. Additionally, the 5CeO_2_/Co_3_O_4_ sensor showed a low detection limit of 300 parts per billion (ppb), a swift response time of 13 s, a moderate recovery time of 48 s, as well as impressive selectivity, reproducibility, and stability over a period of 30 days. In another similar work, a high-quality CO-sensing material composed of porous n-ZnO/p-Co_3_O_4_ NSs was successfully synthesized utilizing a solid rhombic dodecahedron ZIF-67 as a template, followed by a Zn^2+^ etching-hydrothermal-calcination method, and delivered a high response value (*R*_g_/*R*_a_) of 35.4 at 150 °C for 50 ppm CO and exceptional anti-interference capability with a selectivity ratio (S_CO_/S_H2_) of 4.8 [65]. Using a MOF-derived method, Chen et al. [31] directly deposited ZnO nanoflowers onto ceramic substrates and subsequently grew Co_3_O_4_ in situ on the ZnO nanoflowers (Figure 4f). The optimal Co_3_O_4_/ZnO sensor demonstrated an impressive response of 38% to 100 ppm CO at 210 °C (Figure 4g), accompanied by rapid response and recovery times of 38 s and 50 s, respectively. Furthermore, the sensor exhibited exceptional selectivity towards CO, outperforming its selectivity for NH_3_ by a factor of 14 when exposed to 500 ppm of both gases (Figure 4f). Notably, the response remained stable when 100 ppm CO was mixed with 100 ppm of interfering gases such as H_2_, CH_4_, and NH_3_. The outstanding CO-sensing performances of these sensors were attributed to their uniquely constructed heterostructures, which provided a large surface area and abundant active sites, thereby enhancing gas adsorption and reaction kinetics, the synergistic effects arising from the formation of the heterojunctions between two comments, and the abundant oxygen vacancies.

**Figure 5:**
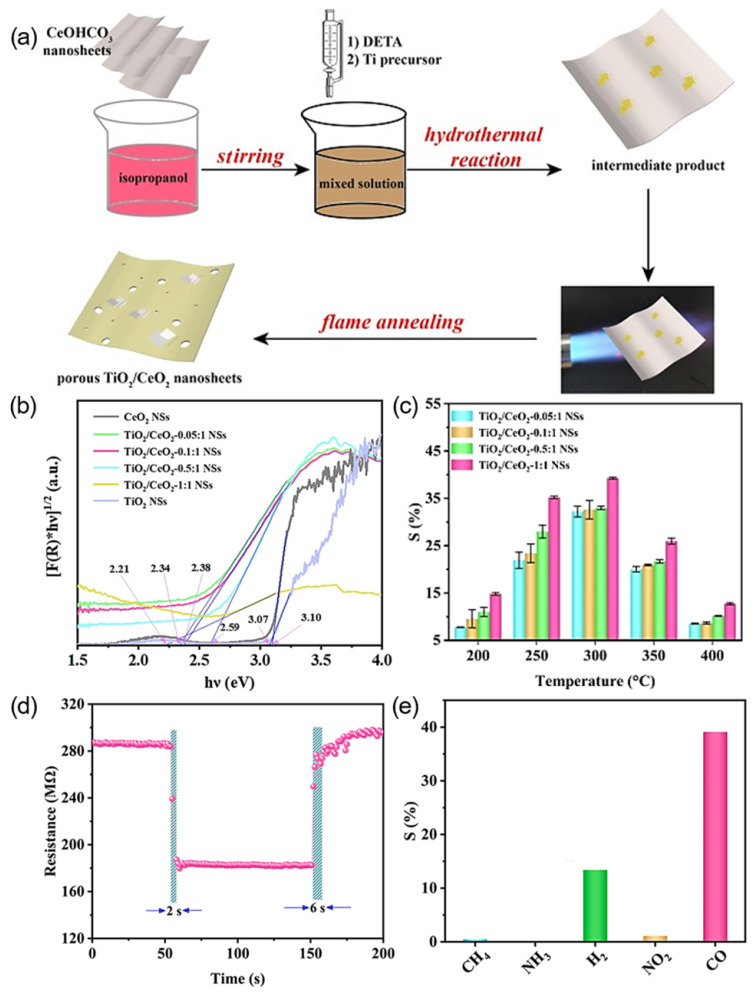
(**a**) Schematic illustration of preparation of TiO_2_/CeO_2_ NSs. (**b**) Band gap of TiO_2_/CeO_2_ NSs. Gas-sensing performances of TiO_2_/CeO_2_: (**c**) Determination of optimal operating temperature of TiO_2_/CeO_2_ NSs, from 200 °C to 400 °C with an interval of 50 °C, (**d**) response/recovery time of TiO_2_/CeO_2_-1:1 NSs towards 500 ppm CO at 300 °C and (**e**) selectivity of TiO_2_/CeO_2_-1:1 NSs towards 500 ppm CH_4_, NH_3_, H_2_, CO and 5 ppm NO_2_ at 300 °C.

In addition to utilizing MOFs as precursors in conjunction with the hydrothermal calcination approach to synthesize heterojunctions, alternative precursors paired with diverse methodologies can likewise be employed for the preparation of such interfaces. For example, Chen et al. [71] fabricated porous TiO_2_/CeO_2_ nanocrystals with varying TiO_2_ concentrations through a combination of a hydrothermal process and flame annealing (Figure 5a). The TiO_2_-CeO_2_ heterojunction, by narrowing the bandgap of the composite system (Figure 5b), enhanced electron transfer efficiency and confers exceptional gas-sensing capabilities to the TiO_2_/CeO_2_ NS. The response value of the optimal TiO_2_/CeO_2_-1:1 NSs (Figure 5c) surpassed that of CeO_2_ NSs by a factor of 9.78 while exhibiting short response and recovery times (2 s/6 s, Figure 5d), superior selectivity towards CO (Figure 5e), and a broad CO detection range spanning from 500 ppb to 5000 ppm [71]. Tang et al. [69] synthesized the p-Co_3_O_4_/n-Co_3_S_4_ heterojunction through a combination of two-phase hydrothermal synthesis and CVD techniques for CO detection [69]. The optimal operational temperature of the sensor was determined to be 200 °C in both air and SF_6_ environments. Notably, the sensor exhibited excellent selectivity towards CO in both conditions, along with robust resistance to moisture and long-term stability [69].

Furthermore, the integration of materials possessing exceptional conductivity, chemical stability, and high surface-to-volume ratios, such as graphene, reduced graphene oxide (rGO) [73], carbon nanotubes (CNTs), and polyaniline (PANI), with those exhibiting sensitivity to CO, including metal oxide semiconductors, metal nanoparticles, and polymers, offers a pathway to maximize their functions. This strategic combination and adjustment of the proportion and structural configuration of the composite materials enables the optimization of their response toward CO. For instance, Han et al. [74] reported that nanocomposites formed by depositing Pt nanoparticles on CNT sheets exhibited superior CO gas-sensing performance compared to pristine CNT sheets. The Pt-CNT sensor demonstrated a rapid response to CO, with a maximum response time of approximately 30 s and a minimum recovery time of approximately 40 s [74]. Additionally, the composite of SnO_2_ nanoparticles with PANI (PANI/SnO_2_) has been demonstrated to improve low-temperature CO-sensing performance [75]. These findings show the potential of hybrid materials in advancing CO detection technologies.

### 3.2. Nanostructured Design

**Figure 6:**
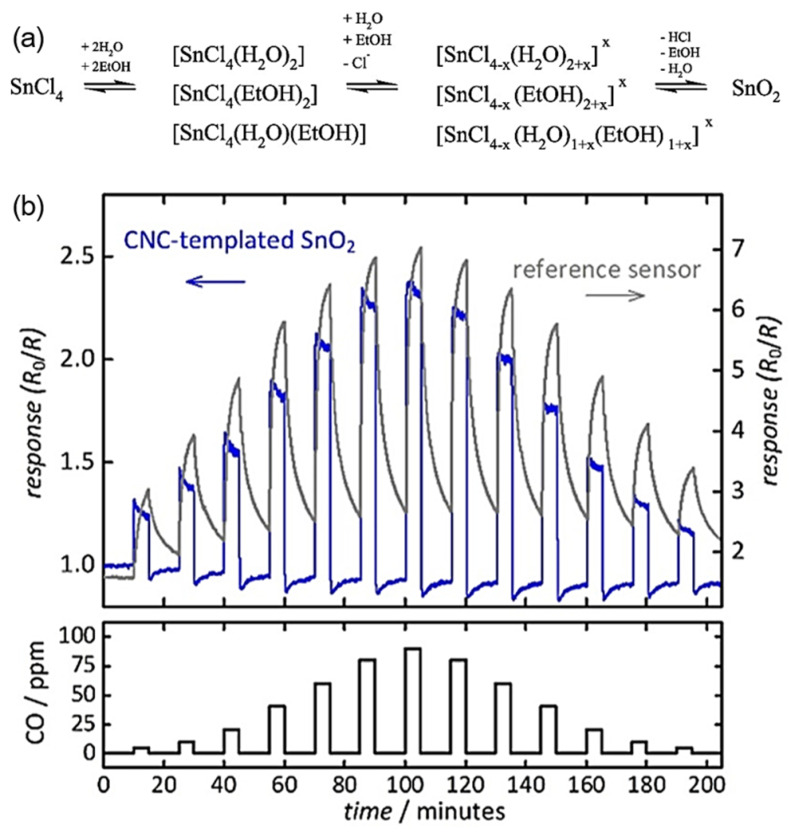
(**a**) Key elements of the mechanism of SnO_2_ formation via sol–gel processes when tin tetrachloride is dissolved in ethanol and water [72]. (**b**) Performance of the CNC-templated tin reference SnO_2_-based commercial sensor UST GGS 1330 [72].

By controlling the nanostructures of sensitive materials, including NSs, nanowires, nanorods, nanoparticles, nanoflowers, and porous structures, one can manipulate their specific surface area and charge distribution to favor the gas adsorption, reaction, and diffusion [76,77,78,79,80]. Consequently, this optimizes their responsiveness to CO. Furthermore, the design of such nanostructures enhances the stability and repeatability of sensors, as nanoscale materials frequently exhibit superior anti-aging and anti-poisoning properties. In-home air-quality monitoring devices and sensors with nanostructured materials like porous SnO_2_ thin films can quickly and accurately detect low-concentration CO released from malfunctioning gas stoves. In recent years, various synthetic methodologies have been employed to prepare these diverse nanostructures. Ivanova et al. [72] introduced a method for synthesizing porous SnO_2_ thin films utilizing cellulose nanocrystals (CNCs) as a universal templating agent (Figure 6a). Their study revealed that the structural attributes of the CNC-templated SnO_2_ thin films were strongly contingent upon the precursor composition. By fine-tuning the precursor mixture, they successfully fabricated high-porosity thin films with pore diameters spanning from 10 to 20 nm, exhibiting specific surface areas ranging from 46 to 64 m^2^ g^−1^. The assembled CNC-templated SnO_2_ sensor demonstrated remarkable sensitivity to ppm-level concentrations of CO, surpassing the commercial sensor (Figure 6b) while exhibiting low cross-sensitivity to humidity. The ultra-thin porous membrane facilitated rapid gas accessibility, resulting in rapid transmission kinetics. Specifically, the response to analyte gases and signal attenuation following gas exposure occurred within seconds, outperforming standard SnO_2_-based CO sensors [72].

Several studies have shown that directly synthesized three-dimensional (3D) nanoflowers, comprised of a substantial number of well-ordered one-dimensional (1D) and two-dimensional (2D) nanostructures, offer ample space for the adsorption and desorption of gas molecules. These structures also introduce a higher density of active sites on the surface of the materials, leading to a significant enhancement in the response of sensors. Furthermore, some research has indicated that nanorods may exhibit superior CO-sensing performance compared to nanospheres, attributed to their unique morphological characteristics. Amoresi et al. [81] elucidated a correlation between the particle shape/size and various factors, including the types of surface species, carrier concentration, vacancy density, and local structural atomic ordering based on their investigation on Ni_2_O_3_-decorated CeO_2_ nanoparticles as a CO sensor. Both experimental data and density functional theory (DFT) analyses consistently demonstrated that the rod-like morphology offered enhanced CO sensor performance in comparison to the spherical morphology. Li et al. [30] found that compared to the nanospheres, CeO_2_ nanorods exhibited an improved H_2_ gas-sensing performance with a high selectivity to H_2_ over CO.

Furthermore, the achievement of selective response to diverse gases can be facilitated by constructing multilayer sensitive layer structures. Specifically, integrating a layer of material exhibiting high sensitivity to CO with another layer possessing selectivity towards other interfering gases can markedly enhance the CO selectivity of sensors. Utilizing microstructure design methodologies, which encompass advanced microfabrication techniques such as photolithography and etching, enables the precise preparation of microstructures, including micropores and microchannels, on sensing materials.

### 3.3. Surface Modification

**Figure 7:**
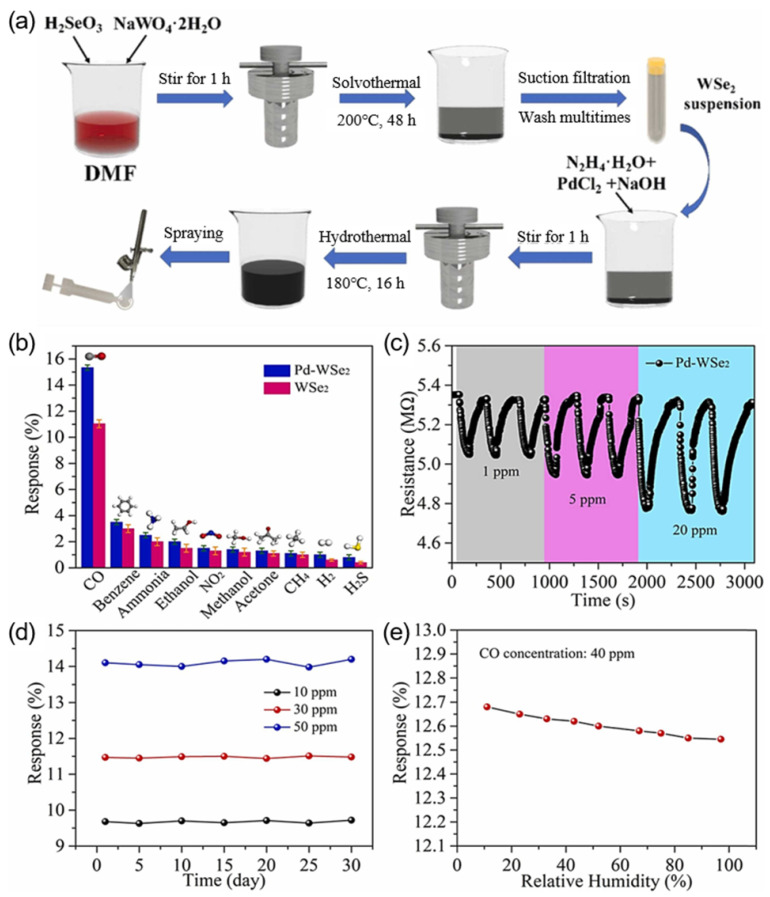
(**a**) Hydrothermal route for preparing Pd-WSe_2_ film sensor [66]. (**b**) Selectivity of Pd-WSe_2_ sensor towards CO and various interfering gases under the same concentration of 100 ppm [66]. (**c**) Repeatability test of Pd-WSe_2_ composite sensor towards 1, 5 and 20 ppm CO gas [66]. (**d**) Long-term stability test of Pd-WSe_2_ composite sensor towards 10, 30 and 50 ppm CO gas [66]. (**e**) Response of Pd-WSe_2_ composite towards 40 ppm CO gas under different relative humidity at room temperature [66].

The introduction of specific functional groups or catalysts onto the surface of sensitive materials can modify their surface chemical properties [82,83], thereby improving their abilities to adsorb and catalyze the oxidation of CO. For instance, depositing a catalyst layer, such as palladium (Pd) or platinum (Pt), onto the surface of sensitive materials through techniques such as CVD or solution impregnation can appreciably enhance their sensitivity to CO. Numerous studies have reported that when precious metals like Pd are loaded onto 2D transition metal dichalcogenides such as MoX_2_ and WX_2_ (where X = S, Se, and Te), the sensing performance for CO surpasses that of other metal loadings [66,84]. Zhang et al. observed that the Pd-WSe_2_ hexagonal nanosheet thin film, synthesized via the hydrothermal method (Figure 7a), exhibited exceptional sensing performances toward CO, encompassing high sensitivity (Figure 7b), outstanding repeatability (Figure 7c), favorable selectivity (Figure 7b), robust stability (Figure 7d) and low humidity dependence (Figure 7d). At room temperature, the response and recovery times were determined to be 52 s and 97 s, respectively, with a response of 9.25% at a CO concentration of 5 ppm (Figure 7). DFT analyses confirmed that noble metal Pd-doping exhibited superior sensing performance for CO compared to doping with other metals [66].

**Figure 8:**
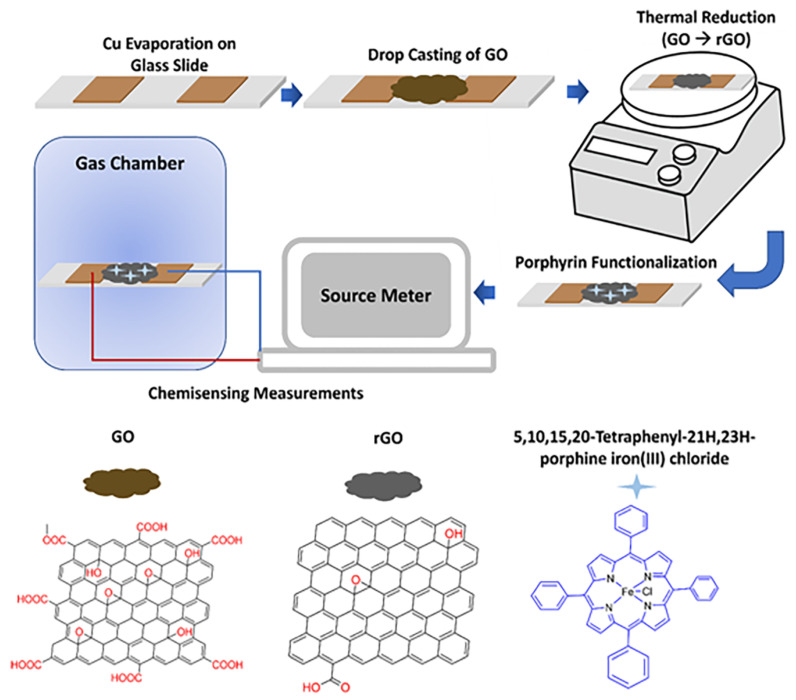
Schematic representation of experimental procedures along with the molecular structures of rGO and FeTPP [67].

Metalloporphyrin-functionalized rGO, denoted as MOEP-rGO (where M = Zn, Ru, or Fe, and OEP = octaethyl porphyrin) [67,85,86], has demonstrated remarkable sensitivity and selectivity towards CO. This functionalized material facilitates enhanced CO adsorption via van der Waals forces and coordination interactions with the central metal ions. The 2D nanostructure of rGO facilitates rapid charge conduction through covalent bonds. Additionally, the core metal ions and peripheral ethyl groups of the metalloporphyrins contribute to the enhancement of the sensor’s sensitivity and selectivity towards CO. For example, Bodkhe et al. [86] reported a highly efficient chemiresistive sensor based on FeOEP-functionalized rGO (rGO/FeOEP), which exhibited high sensitivity, selectivity, stability, repeatability, and reproducibility for CO, with a limit of detection of 1 ppm. Shirsat et al. [67] reported iron tetraphenylporphyrin (FeTPP)-functionalized rGO (FeTPP@rGO) chemiresistive sensors for CO detection (Figure 8). The device exhibited response and a recovery time of 60 s and 120 s, respectively, with a limit of detection of 2.5 ppm.

### 3.4. Doping and Alloying

**Figure 9:**
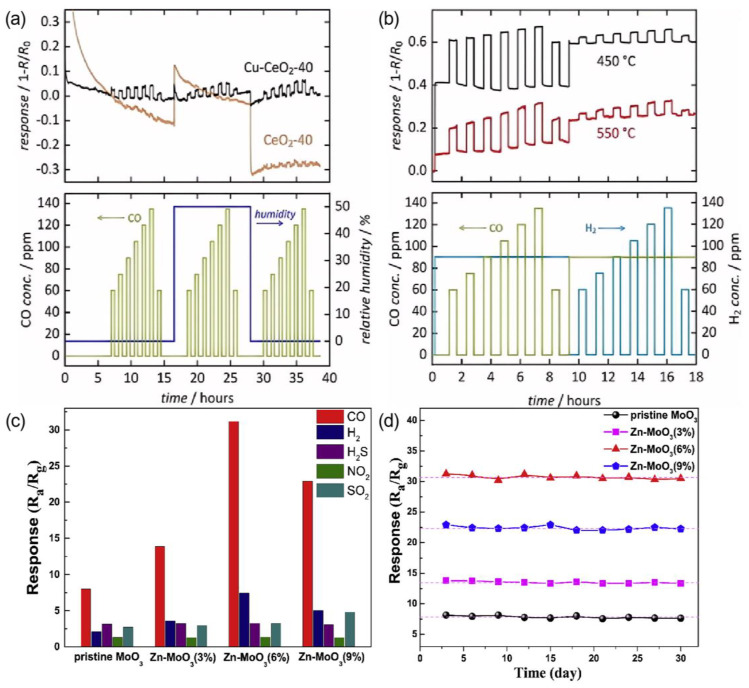
(**a**) Response of CeO_2_-40 and Cu–CeO_2_-40 to CO gas in variable concentration under dry and humid (50% r.h.) conditions at 600 °C. (**b**) Response of Cu–CeO_2_-40 to CO and H_2_ gases offered simultaneously in variable concentrations at 450 and 550 °C under dry conditions [87]. (**c**) Selectivity of sensors based on Z-0, Z-3%, Z-6% and Z-9% to 50 ppm gases and (**d**) repeatability of sensors to 50 ppm CO at optimum temperatures [68].

The incorporation of additional elements or the formation of alloy structures within sensing materials can modify their electronic configurations, ultimately optimizing their selectivity towards CO. The specific dopants employed depend on the nature of the substrate. Baier et al. [87] observed that Cu-modified CeO_2_ demonstrated improved CO-sensing capabilities compared to the pristine CeO_2_ due to the increased specific surface area, uniform porosity, and multi-valent states of metal ions (Cu^+^, Cu^2+^ and Ce^3+^, Ce^4+^). This sensor exhibited a more pronounced response to CO compared to H_2_ and displayed reduced cross-sensitivity to humidity (Figure 9a,b) [87]. The high selectivity was attributed to the stronger tendency of CO than H_2_ to occupy the active sites. Wang et al. [68] synthesized Zn-doped MoO_3_ hierarchical microflowers exhibiting an outstanding CO-sensing performance utilizing a facile one-step hydrothermal synthesis approach. The sample doped with 6 mol% Zn displayed optimal detection characteristics for 50 ppm CO, achieving a response value of 31.23, which was four times higher than that of pristine MoO_3_ at a relatively low temperature of 240 °C. It also exhibited excellent CO selectivity (Figure 9c) and repeatability (Figure 9d). The electronic properties calculations revealed the beneficial effect of Zn-doping on the adsorption capacity of MoO_3_ towards CO molecules [68].

Various metal dopants, including Cd [88], Mo [27], etc., have been experimentally validated to improve the gas-sensing capabilities of Co_3_O_4_. A yeast-shaped Mo-Co_3_O_4_ material from a Co-MOF precursor was successfully fabricated by Hussain et al. [27] via a solvothermal-annealing method for a CO-detection gas sensor. The introduction of Mo dopants into the Co_3_O_4_ material and the hierarchical yeast-like nanostructure co-induced the improved sensing capabilities of Mo-Co_3_O_4_ compared to the pure Co_3_O_4_. At 200 °C, 2 mol% Mo-Co_3_O_4_ reached a high response level of about 136 at 100 ppm CO concentrations, which was about 50.4 times higher than pure Co_3_O_4_. Additionally, the Mo-doped material demonstrated a remarkably low detection limit, exceptional reproducibility, prolonged stability, favorable selectivity, and fast response and recovery times of 78.5 and 55.3 s, respectively.

**Figure 10:**
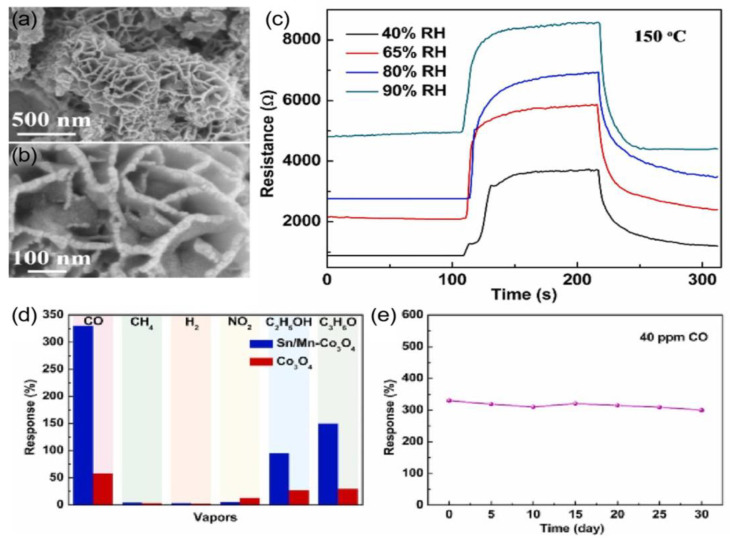
(**a**,**b**) FESEM images of Sn/Mn-Co_3_O_4_. (**c**) Real-time resistance changes curves of Sn/Mn-Co_3_O_4_ toward 40 ppm CO at 150 °C under different RH conditions. (**d**) Selectivity test for Sn/Mn-Co_3_O_4_ toward 40 ppm different gases at 150 °C. (**e**) Long-term stability of Sn/Mn-Co_3_O_4_ toward 40 ppm at 150 °C [70].

In addition to single-doping, co-doping with two different hetero-elements could often further promote the sensing properties due to their synergistic effect. The experimental findings revealed that the 1 mol% Sn/Mn co-doped Co_3_O_4_ sample with a porous nanosheet structure (Figure 10a,b) demonstrated an enhanced response [(*R*_g_ − *R*_a_)/*R*_a_ = 330% at 40 ppm and 150 °C] towards CO compared to pristine Co_3_O_4_, 1 mol% Sn-doped Co_3_O_4_, and 1 mol% Mn-doped Co_3_O_4_ NSs. Furthermore, the 1 mol% Sn/Mn co-doped Co_3_O_4_ NSs exhibited a low detection limit (500 ppb), selectivity, a low humidity dependence (Figure 10c), good selectivity (Figure 10d), and good stability (Figure 10e) [70]. The generation of electrons that compensate for the substitution of Co^2+^ sites by higher valence state Sn^4+^ subsequently led to a reduction in the hole concentration in the air.

In summary, the enhancement of the performances of chemiresistive CO sensors through the improvement of the sensing material represents a complicated yet effective strategy. This approach encompasses the meticulous selection and design, modification, and structural optimization of the sensing materials utilized within the sensors.

## 4. Construction of a Sensor Array and Improving Gas Recognition Algorithms

While enhancing the properties of sensing materials can indeed promote the performances of CO sensing, these advancements alone may be inadequate to attain the desired selectivity and sensitivity, particularly in scenarios where the concentration of interfering gases surpasses that of CO by a significant margin. Consequently, in recent years, there has been a broad adoption of strategies aimed at optimizing sensor structures and gas recognition algorithms to achieve improved performances [89,90,91]. The integration of diverse gas sensors into a sensor array can facilitate the achievement of high selectivity in recognizing specific gases, such as CO. For more complex scenarios involving gas mixtures, the deployment of sophisticated classification algorithms alongside diverse and highly sensitive gas sensor arrays with exceptional selectivity is indispensable.

**Figure 11:**
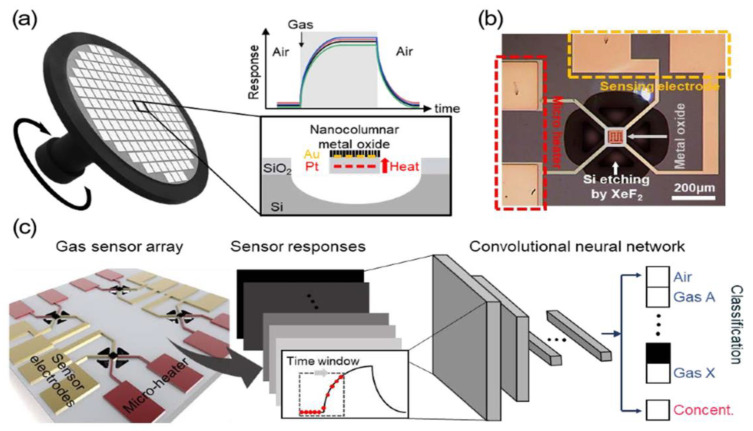
(**a**) Schematic diagram for the fabrication of high batch-uniform semiconductor metal oxide gas sensors using glancing angle deposition (GLAD). (**b**) Microscopic image of the suspended microheater platform-based SMO gas sensors whose sensing material is with nanocolumnar In_2_O_3_ thin films deposited through GLAD. (**c**) Real-time classification and regression of target gases by the CNN-based analysis of gas-sensing data [102].

In comparison to recent research endeavors, the algorithms currently utilized in gas sensing encompass principal component analysis (PCA) [92,93], linear discriminant analysis (LDA) [94,95], support vector machines (SVM) [17,96], artificial neural networks (ANN) [97,98], and convolutional neural networks (CNN) [99,100]. Applying a thermal gradient to the single nanowire and combining its responses at five different working temperatures, Tonezzer [101] successfully distinguished seven different gases (CO, H_2_, NO_2_, ammonia NH_3_, acetone C_3_H_6_O, ethanol, and toluene) and measured their concentrations using PCA and SVM. Kang et al. [102] employed a CNN algorithm in conjunction with a metal oxide semiconductors sensors array (Figure 11), comprising SnO_2_, In_2_O_3_, WO_3_, and CuO, and successfully classified the target gases, including CO, NH_3_, NO_2_, CH_4_, and C_3_H_6_O, in the mixed gases. CNN consists of convolutional layers, pooling layers, and fully-connected layers. The convolutional layers extract features from sensor array data, the pooling layers reduce the data dimension, and the fully connected layers classify gases to determine their types and concentrations. The input data of the network were the sensor response features that contained the information from the transient region using the time window, and the output data of the network were both the gas types and concentration values of the target gases (Figure 11c).

**Figure 12:**
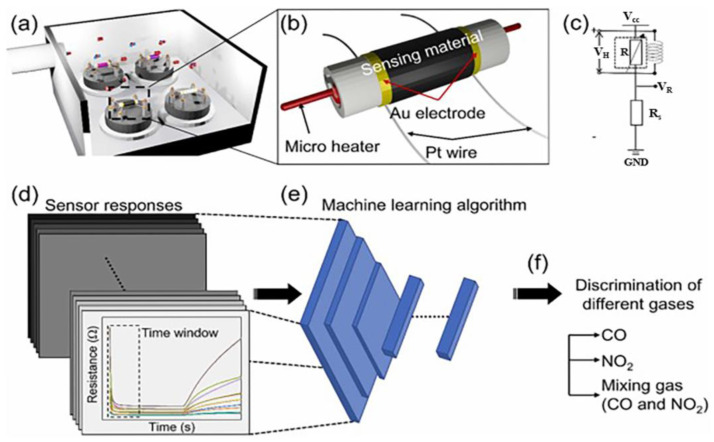
Schematics of a (**a**) gas sensor array, (**b**) fabricated gas sensor, (**c**) voltage divider circuit and heater connection, (**d**–**f**) machine learning-assisted classification and regression of CO and NO_2_ and their mixtures [103].

To further enhance classification accuracy, the dynamic behavior of these sensor arrays can be leveraged effectively, allowing for the elimination of redundant feature extraction steps and facilitating the development of faster pattern recognition systems. In vehicle exhaust detection stations, sensor arrays combined with machine-learning-based gas recognition algorithms, such as the combination of a metal oxide semiconductor sensor array and a CNN algorithm, can accurately identify and quantify CO in vehicle exhaust. This helps monitor vehicle emissions and ensure compliance with environmental regulations. For example, Kim et al. [103] first employed PCA to investigate the discriminating capability of the sensor array and then used the classical ANN-, multilayer perceptron (MLP)-, and CNN-based models to classify various gases and mixtures of CO and NO_2_ in both dry and humid environments by Au-ZnO, Au-SnO_2_, Pt-SnO_2_, and In_2_O^3^-based materials (Figure 12). Among these algorithms, MLP is a basic neural network with input, hidden, and output layers. In gas sensing, the input layer receives data, the hidden layers process the data, and the output layer gives gas classification and concentration predictions. They found that the PCA-assisted MLP model predicted the concentrations of individual gases and mixtures more precisely. Compared to the CNN-based regression, the PCA-assisted MLP model yielded the lowest mean squared error during concentration estimation.

In addition, bivariate sensors, capable of generating either fully or partially distinct 2D response signals from a single sensing element, offer a viable alternative to sensor arrays. Over the past few decades, numerous bivariate sensors, grounded in diverse principles, have been devised for the discrimination and identification of various gaseous species [91]. Within this category, chemiresistive-potentiometric (C-P) bivariate sensors have emerged as highly appealing options for the precise detection and identification of target gases due to their cost-effectiveness, efficiency, and compact footprint [5]. Zhang et al. [104] have fabricated C-P bivariate sensors for the detection and identification of CH_4_ and CO by growing one-dimensional ZnO nanorods in situ as the sensing electrodes on the surface of a Ce_0.8_Gd_0.2_O_1.9_ electrolyte. A total of four C-P sensors were developed by varying the hydrothermal growth duration of the ZnO nanorods. By utilizing an array comprising the ZnO-1.0 and ZnO-1.5 sensors, the research achieved 100% accuracy in differentiating five distinct gases, including CH_4_, CO, and three gas mixtures [104].

## 5. Conclusions and Outlook

The development of high-performance CO sensors is essential for safeguarding human lives. Chemiresistive sensors, a prevalent technology in gas detection, exhibit advantages such as high sensitivity, rapid response times, simplicity in structure, and cost-effectiveness. However, they also possess drawbacks, including limited selectivity and poor stability. To address these challenges, a series of strategies are commonly utilized, including the optimization of sensor materials, sensors, gas recognition algorithms, and so on.

Optimizing sensing materials through techniques like heterostructure construction, doping, and surficial functionalization is crucial for enhancing CO-sensing performance. Experimental validation has confirmed that various metal dopants, including Sn [69], Mn [69], Cd [88], Mo [27], and Ag [105], can enhance the gas-sensing capabilities of Co_3_O_4_. In addition to single-doping, co-doping with two different hetero-elements often further enhances sensing properties due to synergistic effects. The use of metals with multi-valence states may introduce point defects near substitution sites and offer more opportunities for surface chemical reactions, potentially improving CO-sensing performances. For instance, Sn/Mn co-doped Co_3_O_4_ exhibits improved sensing properties towards CO compared to pristine Co_3_O_4_ due to oxygen vacancies induced by Sn^4+^ and Mn^2+^/Mn^3+^ co-doping, a high Co^3+^ ratio, and a high specific surface area [69]. To enhance selectivity towards CO, especially in environments containing H_2_, which exhibits similar response behavior, it is crucial to meticulously select the appropriate component. Fabricating an active metal center with stronger CO adsorption than H_2_ adsorption could be a viable strategy, as exemplified by Cu-CeO_2_ [86].

Beyond improving CO selective sensing by enhancing sensitive material properties, sensor performance can be further promoted by optimizing sensor structure and gas recognition algorithms. Specifically, sensor optimization strategies include integrating multiple sensors into an array and utilizing multimodal recognition [104,106,107,108]. A combination of sensor arrays interfaced with a machine-learning platform could yield an effective model for the real-time application of high-accuracy CO sensors. However, attention must be given to the issue of overfitting during the process of machine learning, especially when dealing with a limited amount of experimental data. A potential solution for achieving accurate predictions could involve a combined calculation approach, including machine learning [103,109,110], DFT [111,112,113,114,115,116,117], and Monte Carlo simulations [118].

The chemiresistive CO sensors, while promising, face practical limitations in cost. The costs associated with sensing materials escalate due to complex material synthesis processes and the utilization of costly precious metals for surface modifications. Additionally, the expenses for sensor arrays and algorithms increase because of the requirement for multiple sensors and substantial computing resources necessary for algorithm training.

In the future, chemiresistive CO sensors will evolve towards intelligence, miniaturization, multifunctionality, high precision and stability, low power consumption, and environmental protection. These trends will propel continuous advancements in sensor technology and expand application fields, providing more reliable safeguards for life safety and environmental protection. Taking the implementation plan for SnO_2_-based CO sensors as an example, fundamental research and material optimization are initially performed to attain a heightened response (Δ*R*/*R*_0_ > 50% at 100 ppm) to CO at room temperature or below 150 °C while surpassing the selectivity of conventional SnO_2_ sensors. Subsequently, sensor prototype development and performance verification will be carried out for miniaturization design, and a laboratory grade SnO_2_-based CO sensor prototype will be launched with a response time of <30 s, a recovery time of <1 min, and a lifespan of >1 year. Next, environmental adaptability and pilot testing will be conducted to preliminarily enter the industrial safety monitoring and smart home market through industrial-grade reliability testing (such as IEC 60068). Finally, we aim to achieve large-scale production and commercialization.

## Figures and Tables

**Table 1:** Performances comparison with reported CO sensors.

Materials	Concentration (ppm)	Response	Temperature (°C)	Response Time/Recovery (s)	Limit If Detection (ppm)	Ref.
Pd-WSe_2_	5	9.25 ^d^	RT	52/97	NA	[66]
FeTPP@rGO	7.5	2.5	RT	60/120	2.5	[67]
Co_3_O_4_/ZnO	10	38% ^c^	210	38/50	NA	[31]
Zn-doped MoO_3_	50	31.23 ^e^	240	10/14	NA	[68]
p-Co_3_O_4_/n-Co_3_S_4_	0.5	30 ppm@29.19 ^a^	200	11/7	NA	[69]
Mo-Co_3_O_4_ Nanoparticles	100	136 ^e^	100	78.5/55.3	0.195	[27]
Sn/Mn-Co_3_O_4_ NSs	40	330% ^b^	150	14/NA	0.5	[70]
5CeO_2_/Co_3_O_4_	50	184% ^b^	200	13/48	0.3	[64]
n-ZnO/p-Co_3_O_4_	50	35.4 ^e^	150	NA/NA	NA	[65]
TiO_2_/CeO_2_	500	39% ^d^	300	2/6	NA	[71]
CNC-templated SnO_2_	90	2.4 ^f^	350	NA/NA	NA	[72]

a: (Δ*R*/*R*_0_) × 100%. b: (*R*_g_ − *R*_a_)/*R*_a._ c: *R*_a_/*R*_g_ × 100 %. d: |*R*_a_ − *R*_g_|/*R*_g_ × 100%. e: *R*_g_/R_a_. f: *R*_0_/*R.* NA—Not Available.

## Data Availability

No new data were created or analyzed in this study. Data sharing is not applicable to this article.
